# Turning Discarded Oyster Shells into Sustainable Passive Radiative Cooling Films

**DOI:** 10.3390/polym17030275

**Published:** 2025-01-22

**Authors:** Junghwan Lee, Dae Kyom Kim, Daeyul Kwon, Jeehoon Yu, Jeong Gyu Park, Youngjae Yoo

**Affiliations:** 1 Department of Advanced Materials Engineering, Chung-Ang University, Anseong 17546, Republic of Korea; ppiy555@cau.ac.kr (J.L.); daekyom@cau.ac.kr (D.K.K.); koh1077@cau.ac.kr (D.K.); yujeehoon@cau.ac.kr (J.Y.); 2 PMI BIOTECH Inc., Geoje 53281, Republic of Korea; lena.heo@pmibiotech.com

**Keywords:** discarded oyster shell, calcium carbonate, radiative cooling, cooling performance, marine pollution

## Abstract

Inorganic materials used in passive radiative cooling have achieved a commendable level of performance through synthesis, yet they lack sustainability and environmental friendliness as they do not incorporate recycling. This study developed a novel passive radiative cooling (PRC) film utilizing calcium carbonate extracted from discarded oyster shells (D-CaCO_3_) and polyurethane (PU) as the matrix. This sustainable approach leverages the unique properties of CaCO_3_, such as high solar reflectance and strong infrared emissivity, to achieve significant cooling effects. The PU/D-CaCO_3_ film absorbs only 22% of total solar light and exhibits a high emissivity of 95% in the atmospheric window, achieving temperatures up to 7 °C lower than the surrounding environment under 650 W/m^2^ solar irradiance. Furthermore, field tests were conducted to verify the implementation of our optical strategy by analyzing the optical properties and FDTD simulations. Consequently, the PU/D-CaCO3 film outperformed conventional white paint and pure PU, demonstrating a maximum temperature difference of 7 °C. Additionally, the passive radiative cooling efficiency of the film was verified through theoretical calculations. The oyster-shell-derived CaCO_3_ utilizes waste and contributes to carbon sequestration, aligning with sustainable and eco-friendly goals. This research demonstrates the potential of using marine-derived materials in passive cooling technologies, offering a path to reduce energy consumption and greenhouse gas emissions in cooling applications. The findings highlight the commercial viability and environmental benefits of PU/D-CaCO_3_ films, marking significant progress in passive radiative cooling.

## 1. Introduction

Traditional cooling systems that utilize various refrigerants accounted for approximately 10% of the total power consumption in the United States in 2021 [1]. The resultant greenhouse gas emissions accelerate global warming [2]. Therefore, there is an increasing need for energy-efficient and environmentally friendly cooling technologies [3]. To meet these demands, passive radiative cooling (PRC) technology has been gaining attention [4]. PRC reflects solar heat in the 0.3–2.5 µm UV-vis-NIR spectrum and emits infrared through the “atmospheric window” in the 8–13 µm infrared region into cold outer space (~3 K) [5]. This allows for effective cooling without needing separate cooling devices, enhancing energy efficiency and promoting sustainability by minimizing environmental impact [6]. For effective PRC, structures must simultaneously possess high solar reflectivity and high infrared emissivity. This requirement has prompted the recent proposal of various PRC structures designed to achieve temperatures below ambient under direct sunlight. These include multilayer structures [7,8], metamaterials [9,10,11,12], randomly distributed particle structures [13,14], and porous structures [15,16]. These radiative cooling structures exhibit excellent performance, but their complex design and high production costs have limited widespread application. Significant research on sustainable and eco-friendly passive radiative cooling has been actively conducted. Zhang’s research team proposed a radiative cooling material recycled from waste PS foam [17]. Song et al. also developed a radiative cooling film using a hybrid wood-derived laminates consisting of wood, epoxy resin, and SiO_2_ [18]. Shi et al. reported thin hierarchically micro- and nanostructured poly(vinylidene fluoride-trifluoroethylene) films via crystallinity alteration using an economical and scalable solvent-evaporation-based method [19]. Despite ongoing research on PRC technology using recycled materials, the challenge of expanding industrial applications remains unresolved [20]. In this study, we developed a PRC film using CaCO_3_ extracted from discarded oyster shells (D-CaCO_3_) through a simple process. Generally, conventional processes for obtaining CaCO_3_ powder from the oyster shells were conducted using high-temperature calcination processes near 1000 °C to obtain raw materials and hydrate [21,22]. However, solution processes using dilute HCl solution were adopted to produce high-quality CaCO_3_ in this work, which is novel and facile in contrast to the conventional process. It is an affordable material with high strength and chemical stability. Additionally, due to its high bandgap (5 eV) [23], it has low solar absorption. Experimental results showed that the PU/D-CaCO_3_ film absorbed only 22% of total solar light and exhibited a high atmospheric window emissivity of 95%, achieving temperatures up to 7 °C lower under 650 W/m^2^ solar irradiation. Durable polyurethane as the matrix allowed for long-term use, demonstrating its commercial viability. Using a material extracted from oyster shells to effectively absorb carbon dioxide in marine ecosystems underscores the sustainability of this eco-friendly technology. When oyster shells form, carbon is absorbed and fixed, and after the organism’s death, it can sequester carbon in a permanent form like limestone on the seabed. Thus, shellfish have the potential to act as a biological pump for removing carbon dioxide, known as blue carbon [24]. The radiative cooling film developed in this study is characterized by a simple process, low cost, eco-friendliness, high durability, and high cooling efficiency, providing an effective passive radiative cooling solution based on blue carbon in marine ecosystem research.

## 2. Experimental

### 2.1. Materials

The polyurethane dispersion (PUD) was provided by Aekyung Chemical Co., Ltd. (Seoul, Korea). CaCO_3_ was provided by PMI Biotech Co., Ltd. (Seoul, Korea). The UV absorber was provided by SONGWON Co., Ltd. (Seoul, Korea). The antioxidant was provided by ZIKO Co., Ltd. (Seoul, Korea). Sodium polyacrylate (NaPA) was purchased from Sigma Aldrich Co. (St. Louis, MO, USA). All the distilled water used in the experiment was triple-distilled. Commercial white paint was acquired from Samhwa Paints Co., Ltd. (Seoul, Korea).

### 2.2. Sample Preparation

Here, 20 g of PUD was mixed with 21 g of CaCO_3_, followed by adding the UV absorber, antioxidant, and NaPA. The mixture was stirred in a planetary mixer (rotational speed: 9, revolution speed: 9) for 5 min to remove bubbles. The mixture was cast onto a 10 cm × 10 cm silicon module using a syringe and dried for 24 h. After complete hardening, it was cut into uniform sizes for experiment use. White paint was continuously applied onto a glass plate with a brush to prepare the paint-coated glass, ensuring that the film thickness matched that used in the experiments.

### 2.3. Sample Characterization

The CaCO_3_ used in the film was analyzed using field-emission scanning electron microscopy (FE-SEM). The reflectance of the PRC film in the solar region (0.25–2.5 µm) was measured using ultraviolet–visible–near-infrared (UV-vis-NIR) spectrophotometry (Cary 5000, Agilent Technologies, Palo Alto, CA, USA). The emittance in the mid-infrared wavelength range (2.5 µm to 25 µm) was measured using Fourier-transform infrared (FT-IR) spectrophotometry (iS20, Thermo Fisher Scientific, Waltham, MA, USA), and the diffuse reflectance was measured using an integrating sphere (PIKE Technologies, Fitchburg, WI, USA).

### 2.4. Experimental Setup

An outdoor cooling performance test system was established to measure the cooling performance of the fabricated PRC film. The overall thermal balance included solar radiation absorbed by the PRC film, atmospheric radiation, heat dissipation to deep space through electromagnetic waves, and heat loss due to the temperature difference between the film and the environment. The experimental system was placed on the roof of a laboratory building (Anseong, Korea, 37°33′5.72″ N, 126°56′14.38″ E). The experimental system consisted of a data logger, a test cavity, and a solar power meter. The main test cavity was made of polystyrene foam to reduce heat dissipation, with the outer surface covered with aluminum foil to reflect solar radiation and the top covered with transparent and low-density polyethylene (LDPE) film. The PRC film was composed of a 3 × 3 cm film. The cooling performance of the film was demonstrated by comparing the temperature of the PRC film with that of commercial white paint over several days. Additionally, outdoor atmospheric conditions such as relative humidity and wind speed were obtained from the Metrological Administration.

### 2.5. Calculation of Net Cooling Power

The net cooling efficiency of the PRC film was determined using MATLAB software, which involved simulating the film’s thermal behavior using the energy balance below:

(1) $$P_{net}\left(T\right)=P_{rad}\left(T\right)-P_{Sun}-P_{non,rad}-P_{atm}(T_{amb})$$

where P_rad_(T) denotes the emitted radiance power from the cooling apparatus; P_Sun_ denotes the solar irradiance absorbed by the device; P_atm_(T_amb_) denotes the power absorbed from the atmosphere at T_amb_; and P_non-rad_ represents the non-radiative power absorbed from the surrounding environment [2].

(2) $$P_{rad}\left(T\right)=A\int d\Omega con\theta \int_{0}^{\infty }d\lambda I_{BB}\left(T,\lambda \right)\varepsilon \left(\lambda ,\theta \right)$$

The integral $\int d\Omega =2\pi \int \begin{array}{c} \pi /2\\ 0\; \end{array}d\theta sin\theta$ represents the angular integration across a hemisphere. Planck’s formula provides the spectral emittance of a blackbody at an absolute temperature T.

(3) $$I_{BB}=(2hc^{2}/\lambda^{5})/[e^{hc/(\lambda \kappa ΒT)}-1]$$

Here, h represents Planck’s constant; c denotes the speed of light; κΒ denotes Boltzmann’s constant; and λ denotes the wavelength. The equation below describes the power absorbed by the atmosphere (P_atm_) at the ambient temperature (T_amb_):

(4) $$P_{atm}\left(T_{amb}\right)=A\int d\Omega con\theta \int_{0}^{\infty }d\lambda I_{BB}\left(T_{amb},\lambda \right)\varepsilon \left(\lambda ,\theta \right)\varepsilon_{atm}(\lambda ,\theta )$$

and the atmospheric emissivity is given by

(5) $$\varepsilon_{atm}\left(\lambda ,\theta \right)={1-t\left(\lambda \right)}^{1/cos\theta }$$

The solar power (P_Sun_) absorbed by a radiative cooler is given by the following:

(6) $$P_{sun}=A\int_{0}^{\infty }d\lambda \varepsilon \left(\lambda ,\theta \right)I_{AM1.5}(\lambda )$$

Here, the terrestrial solar irradiance on the surface, represented by the AM 1.5G spectra, is denoted by t(λ)^1/cosθ^, where t(λ) is the atmospheric transmittance at the zenith angle at Mauna Kea.

(7) $$P_{non-rad}={Ah}_{c}(T_{amb}-T_{sam})$$

Considering a steady ambient environment and a solar irradiance of 800 W m^−2^, theoretical cooling power was calculated for different h_c_ values (0, 3, 6, and 9 W m^−2^ K^−1^). The calculation considered the reduction in solar irradiance and the thermal radiation from the LDPE film after placing radiative coolers underneath, assuming the absorption of sunlight by the LDPE film.

## 3. Results and Discussion

### 3.1. Preparation of Discarded Oyster-Shell-Derived CaCO_3_ Film

**Figure 1:**
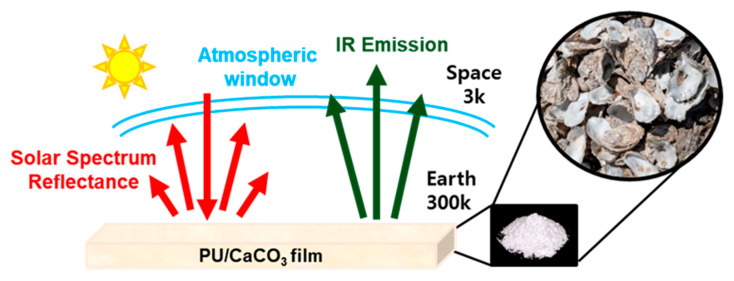
Schematic of radiative cooling principle and radiative cooler structure.

**Figure 2:**
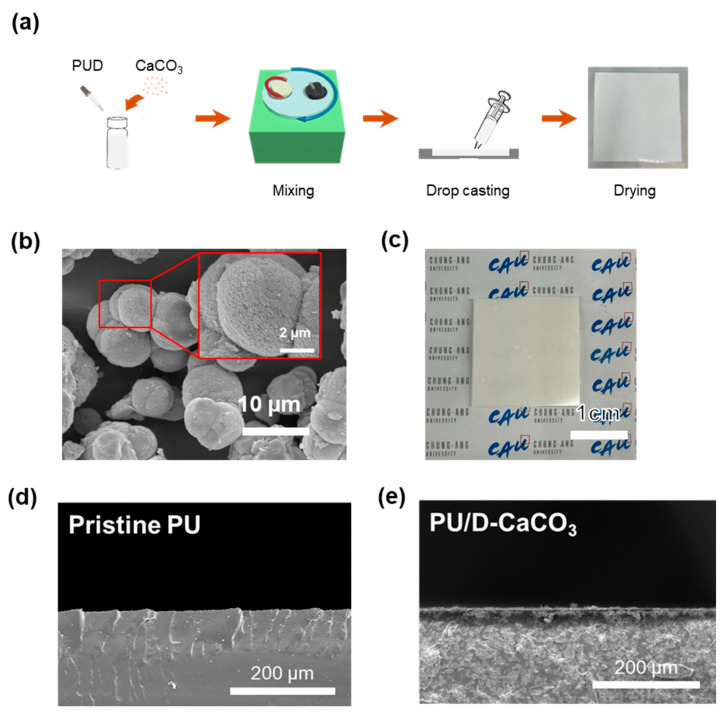
(**a**) Schematic of the PU/D-CaCO_3_ film manufacturing process, (**b**) SEM image of the discarded-oyster-shell-extracted CaCO_3_ (D-CaCO_3_), and (**c**) the passive radiative cooling film. Cross-sectional SEM images of (**d**) pristine PU and (**e**) PU/CaCO_3_ 60 *v*/*v*%. (at 300× magnification).

CaCO_3_ powder shows white color due to its high reflectance in the UV-vis-NIR range, including the visible light spectrum. Additionally, the FT-IR spectrum of CaCO_3_ shows peaks at 744, 876, and 1087 cm^−1^ due to C–O–C bond vibrations, contributing to absorption and emission in the atmospheric window region. Enhancing absorption and emission in this atmospheric window area is crucial for improving radiative cooling performance. The optical properties suitable for passive radiative cooling materials, characterized by low absorption rates and high reflectance in the UV-vis-NIR range, highlight the potential application of waste oyster-shell-derived CaCO_3_. However, the low mechanical properties of CaCO_3_ are insufficient to ensure the sustainable durability of the passive radiative cooling materials. To address this, we applied polyurethane as a binder and radiative cooling polymer, which is a multi-block copolymer with alternating soft and hard segments. This unique structural feature exhibits specific physical properties from soft rubber-like to hard plastics [25,26]. Furthermore, it exhibits good emissivity in the atmospheric window region due to C-N and C-O-C bonds. Figure 1 presents a schematic of the film using CaCO_3_ extracted from waste oyster shells and the principles underlying our passive radiative cooling strategy. Despite using recycled materials, our PU/D-CaCO_3_ film reflects solar heat in the 0.3–2.5 µm range and emits heat in the 8–13 µm infrared range, allowing cooling to temperatures lower than the surrounding environment. Figure 2a is a schematic diagram of the simple manufacturing process of PU/D-CaCO_3_. Using a planetary mixer, we added and mixed polyurethane dispersion, D-CaCO_3_, UV absorber, antioxidant, and sodium polyacrylate. Afterward, we cast a certain amount into a prepared silicon module and slowly air-dried it at room temperature for 24 h to obtain the film. Figure 2b depicts the CaCO_3_ surface where the clustering of nanospheres into micro-sized spherical bodies enhances reflectance due to multiple scattering within particles compared with single-sized spheres. Figure 2d,e are cross-sectional images of pristine PU and PU/D-CaCO_3_, showing that the D-CaCO_3_ particles were well dispersed in the PU matrix. Figure 2c shows the fabricated film, demonstrating sufficiently low transmittance on a white background.

### 3.2. Simulating the Optical Properties of PU/D-CaCO_3_ Film Through the Finite-Difference Time-Domain Method

**Figure 3:**
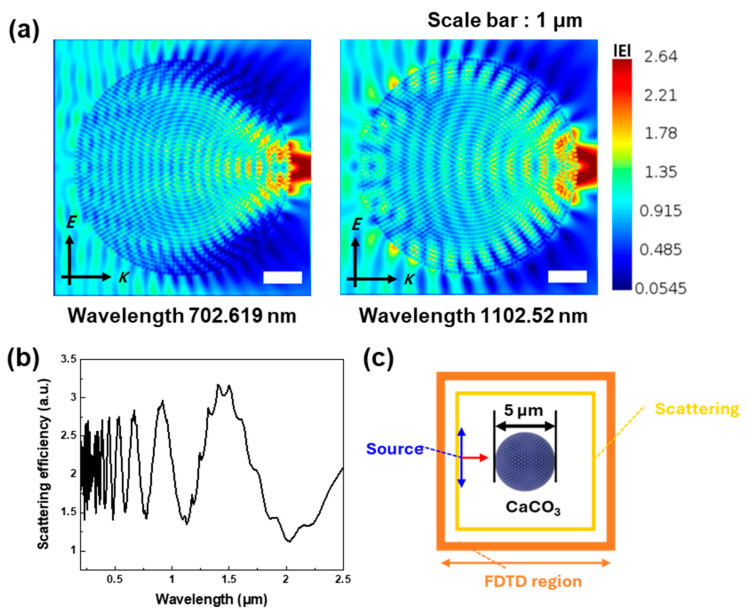
(**a**) Electric field distribution of D-CaCO_3_ particle with 702.619 and 1102.52 nm. (**b**) Scattering efficiency and (**c**) FDTD modeling schematics of D-CaCO_3_.

We previously verified the morphology of particles extracted from the waste oyster shell and modeled the structure of the PRC particles based on this result, as shown in Figure 3c. Consequently, we used PU as the matrix for our PRC film and conducted the simulation with precise spacing arrangements to ensure that the PRC filler particles were positioned at the central focal point. Figure 3a shows the electric field distribution in the FDTD cross-section. A 702 nm wavelength was employed for the electric field distribution because it demonstrates the highest energy in sunlight, which helps identify the backscattering occurring in D-CaCO_3_ particles. Additionally, the scattering of D-CaCO_3_ adjusted at the longer wavelength of 1102.52 µm implies higher total scattering efficiency in the sunlight-illuminated range, increasing the solar reflectance. Figure 3b notably depicts the electromagnetic field distribution where a strong scattering efficiency is observed in the UV-vis range with a scattering efficiency exceeding 1 although the sine curve is not fully developed in the NIR range. This simulation predicts strong reflectance in the UV-vis range and suitable reflectance in the NIR range for the PRC film.

### 3.3. Optical Properties of the PU/D-CaCO_3_ Films

**Figure 4:**
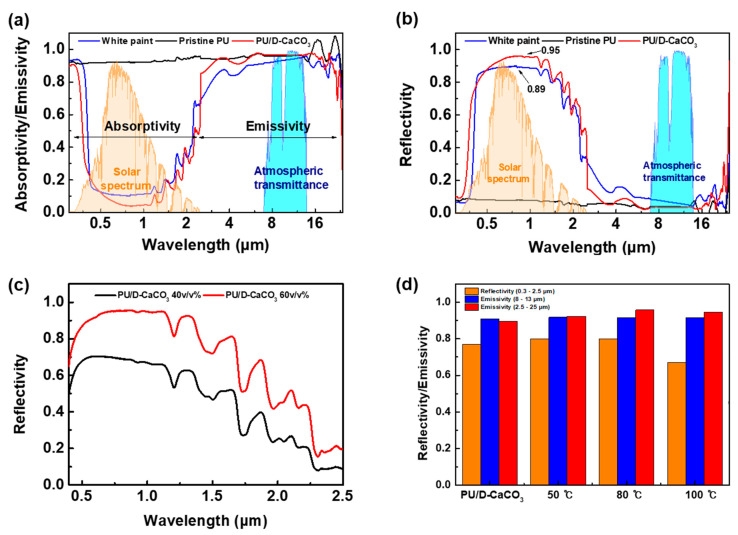
Spectra of (**a**) absorptivity, emissivity, and (**b**) reflectivity of white paint, pristine PU, and PU/D-CaCO_3_. (**c**) Reflectivity of PU/D-CaCO_3_ 40 *v*/*v*% and PU/D-CaCO_3_ 60 *v*/*v*%. (**d**) Thermal stability test of PU/D-CaCO_3_ with different temperatures at room temperature, 50, 80, and 100 °C over 72 h.

We conducted analyses to verify whether our PRC film’s optical properties predicted based on previous simulations were well implemented. The primary film analyzed contained 60 vol% CaCO_3_ compared to polyurethane and had a thickness of 1 mm. Figure 4a reveals the solar absorption and infrared radiation characteristics of PU/D-CaCO_3_, polyurethane, and white paint. The average solar absorption rates of the PU/D-CaCO_3_ and the conventional TiO_2_-based white paint were 22 and 29%, respectively. Furthermore, the average atmospheric window emissivity rate of the PU/D-CaCO_3_ and the conventional TiO_2_-based white paint was 96 and 95%, respectively (Table 1). These results indicate that the differences in solar absorption rate can be attributed to the differences in surface temperature. Figure 4b shows the reflectance characteristics of the PU/D-CaCO_3_ film and white paint. The PU/D-CaCO_3_ film and white paint achieved a maximum solar reflectance of 95 and 89%, respectively. Moreover, the average total solar reflectance of the PU/D-CaCO_3_ film and white paint was 77 and 70%, respectively. According to Kirchhoff’s law of radiation, the sum of absorptance (emittance), reflectance, and transmittance equals 1. Since the film is opaque, the transmittance value was ignored. The PU/D-CaCO_3_ film exhibited a lower absorption rate than the white paint, resulting in higher reflectance in the solar range. The solar range in the UV wavelength regions showed low reflectance by the inherent absorption rate of polyurethane, causing a reflectance loss of about 5% of the total solar range. Figure 4c shows the reflectivity of PU/D-CaCO_3_ with increasing filler contents from 40 to 60 v/v%. As the D-CaCO_3_ content increased, the reflectance in the wavelength ranges from 0.3 to 2.5 μm also increased. To confirm the thermal stability, PU/D-CaCO_3_ was tested at 50, 80, and 100 °C for 72 h (Figure 4d). Consequently, the emissivity showed only minimal changes in the atmospheric window and the broad infrared range, and the average reflectance decreased by about 1.15 times at 100 °C, attributed to the high-temperature effect on the binder, leading to a slight increase in surface roughness. Overall, PU/D-CaCO_3_ demonstrated surface stability and aging resistance, confirming the long-term application potential of eco-friendly D-CaCO_3_ particles for external use in buildings.

### 3.4. Outdoor Field Tests

**Figure 5:**
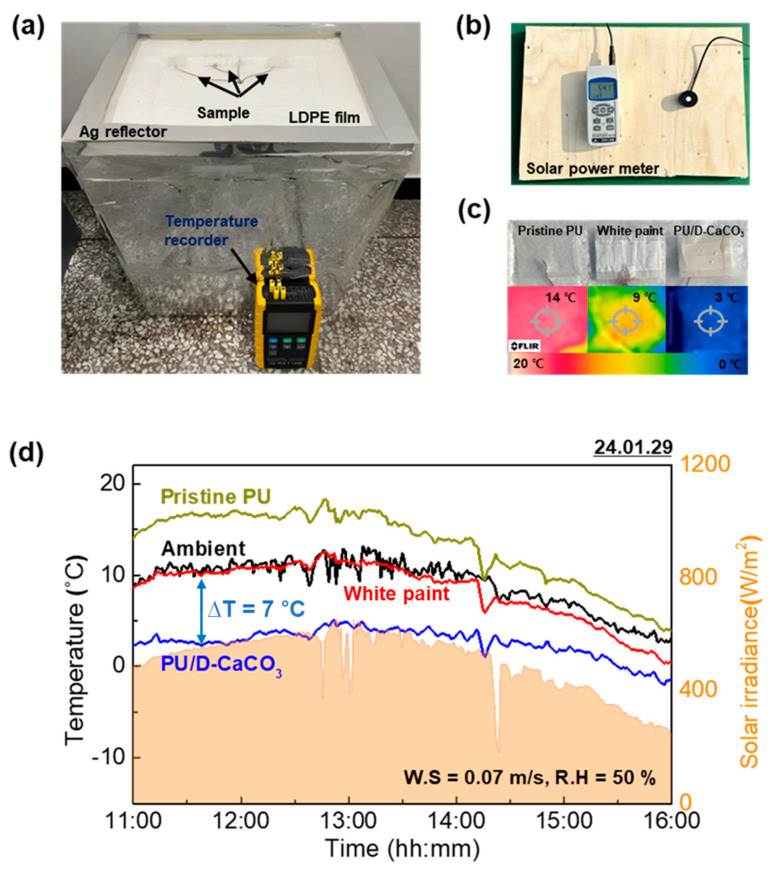
(**a**) Outdoor measurement system, (**b**) solar power meter for radiative cooling, (**c**) photographic and thermal IR images of the radiative coolers during the outdoor experiment (pristine PU: 19 °C, white paint: 21 °C, PU/D-CaCO_3_: 14 °C), and (**d**) radiative cooling performance of PU/D-CaCO_3,_ white paint, and pristine PU in terms of temperature at 11:00–16:00.

Figure 5a displays the setup for the outdoor experiment. The samples used were white paint, pristine PU, and PU/D-CaCO_3_. A thermocouple was attached to the bottom of the samples to measure surface temperatures. To minimize the impact of solar irradiation, a silver reflector was attached to a polystyrene foam box to reflect light. Temperatures were recorded using a temperature recorder at one-minute intervals. An LDPE film covered the samples to reduce the influence of ambient temperature. Figure 5b depicts the pyranometer used for temperature measurements during the experiment. Figure 5c displays a thermal-camera-captured image of the samples, visually demonstrating the temperature differences and highlighting the superior cooling performance of our PU/D-CaCO_3_ with a 3 °C difference depicted in blue compared to other samples. Figure 5d presents the results of the daytime outdoor experiment conducted on 29 January 2024, in Anseong-si, Gyeonggi-do (latitude: 37.01, longitude: 127.28). Wind speed and humidity data were obtained from the Korea Meteorological Administration. While the white paint showed only a marginal difference from the ambient temperature, PU/D-CaCO_3_ demonstrated a maximum temperature difference of 7 °C under an irradiance of 650 W m^−2^, confirming that integrating D-CaCO_3_ into conventional PU can provide a maximum cooling efficiency of up to 13 °C and proving our particles to be successful eco-friendly passive radiative cooling agents. The thickness, IR emissivity, and temperature drop of recently developed polymer composites for PRC are summarized in Table 2. Among them, our PU/D-CaCO_3_ composite exhibited the highest performance values compared to other composites [27,28,29,30,31,32].

### 3.5. Cooling Power Calculation

**Figure 6:**
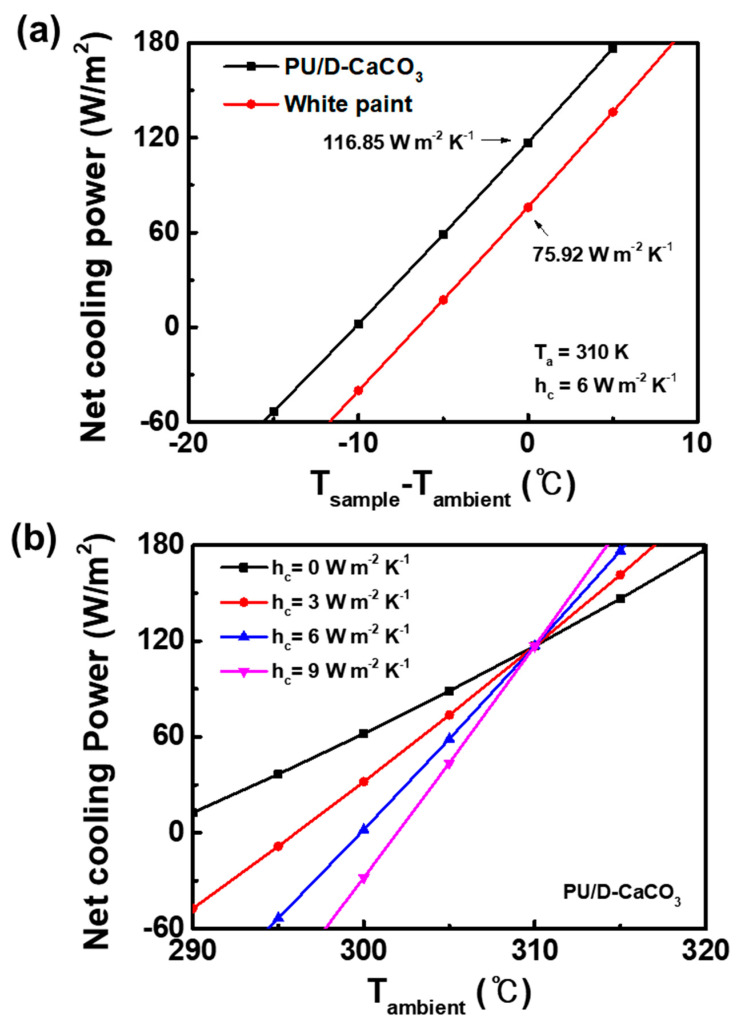
Theoretical cooling power of (**a**) white paint and PU/D-CaCO_3_ at different temperatures, (**b**) theoretical cooling power of PU/D-CaCO_3_ as a function of the non-radiative heat transfer coefficient.

Using MATLAB, we quantified the cooling performance by calculating the theoretical net cooling power of the PU/D-CaCO_3_ film. We assumed various non-radiative heat transfer coefficients of 0, 3, 6, and 9 W m^−2^·K^−1^, as shown in Figure 6b, and calculated the cooling power under 800 W/m^2^ solar radiation and a temperature of 310 K. In Figure 6a, the non-radiative heat transfer coefficient was set at 6 W m^−2^·K^−1^, representing the typical heat transfer coefficient of air. The theoretical cooling power showed values of approximately 116.85 W m^−2^·K^−1^ and 75.92 W m^−2^·K^−1^, representing a cooling efficiency of approximately 1.55 times higher. According to Planck’s law, the radiative energy of the PRC film depends on the surface temperature; thus, as the ambient temperature rises, the radiative energy also increases.

## 4. Conclusions

In this study, we successfully developed a novel PRC film by combining CaCO_3_ extracted from discarded oyster shells with PU. The PU/D-CaCO_3_ film demonstrated low solar absorption and high emissivity in the atmospheric window region, leading to significant cooling effects. Experimental results indicated that this film achieved temperatures up to 7 °C lower than the surrounding environment under 650 W/m^2^ solar irradiance. This performance exceeds traditional cooling methods and demonstrates the potential to significantly reduce energy consumption and greenhouse gas emissions. Outdoor field tests revealed that the PU/D-CaCO_3_ film exhibited superior cooling performance compared to conventional white paint and pure PU. PU/D-CaCO_3_ achieved a maximum temperature difference of 7 °C in these tests, demonstrating its high efficiency under real-world conditions. Theoretical calculations using MATLAB further confirmed the film’s effective cooling potential, implying the commercial viability of the PRC films. This research emphasizes the importance of developing environmentally friendly and sustainable materials. The CaCO_3_ extracted from oyster shells utilizes the concept of blue carbon in marine ecosystems, acting as a biological carbon pump and contributing to environmental protection and sustainable development goals. Compared to traditional cooling systems, this approach can significantly reduce energy consumption and greenhouse gas emissions, reducing the carbon footprint. In conclusion, this study advances passive radiative cooling technology and presents an innovative approach to improving energy efficiency in an environmentally friendly and sustainable manner. It holds great potential to replace traditional cooling systems and contributes to exploring broader applications and commercialization possibilities in the future.

## Figures and Tables

**Table 1:** Comparison of average absorptivity and emissivity rate of PU/D-CaCO_3_ and white paint.

Sample	Average Absorptivity (0.3–2.5 µm)	Average Emissivity (8–13 µm)
PU/D-CaCO_3_	0.22	0.96
White Paint	0.29	0.95

**Table 2:** Polymer composites for PRC and their cooling performance.

Materials	Thickness (um)	IR Emissivity (%)	Temperature Drops (°C)	Refs.
Porous PCA/ SiO_2_ spheres	150	0.95	6.2	[27]
PDMS/glass bubbles	750	0.85	5.3	[28]
Nano-PE/ SiO_2_	840	90	6.1	[29]
PDMS/ZnO	/	81	7.5	[30]
PU/SiO_2_ fibrous membrane	/	94.9	5.4	[31]
SiO_2_-PVA meta fiber	336	90	6	[32]
PU/D-CaCO_3_	1000	96	7	This work

## Data Availability

Data available upon request (due to privacy).
